# Factors influencing chronic disease self-management behaviors: a national multilevel analysis in China

**DOI:** 10.3389/fpubh.2025.1712419

**Published:** 2026-01-07

**Authors:** Kai Li, Yufan Chen

**Affiliations:** 1 School of Public Administration, Hohai University, Nanjing, Jiangsu, China; 2 School of Psychology, South China Normal University, Guangzhou, China

**Keywords:** chronic disease self-management, medical and nursing resources, physicians per 1000 population, national survey, China

## Abstract

**Objective:**

This study employed multilevel analysis to examine the impact of the prefecture-level city level variable (number of physicians per 1,000 population) on chronic disease self-management behavior (CDSMB) among Chinese adults, while also investigating the effects of individual-level control variables on CDSMB.

**Methods:**

A cross-sectional survey of 1,916 adults across 120 cities, using data from the “Psychology and Behavior Investigation of Chinese Residents” (PBICR), evaluated Chronic Disease Self-Management Behavior (CDSMB) via CDSMS. Key predictors included individual-level control variables: health literacy (HLS-SF12), self-efficacy (NGSES), family health (FHS-SF), and the prefecture-level city level variable of physicians per 1,000 population. Hierarchical linear modeling (HLM) was used to analyze the nested data, with adjustments for demographic covariates.

**Results:**

Vulnerable groups (older adults, rural residents, low-income, education individuals) exhibited significantly poorer self-management (*p* < 0.001). At the individual level control variables, health literacy (*β* = 3.075, *p* < 0.001) and self-efficacy (*β* = 1.891, *p* < 0.001) positively predicted CDSMB, while family health showed an unexpected negative effect (*β* = −1.784, *p* < 0.001). At the prefecture-level city level, physicians per 1,000 population (*β* = 0.077, *p* < 0.05) explained 17% of between prefecture-level city level variance in CDSMB.

**Conclusion:**

Based on the findings highlighting the influence of physicians per 1,000 population and individual-level control variables on chronic disease self-management, targeted optimization of medical resource allocation and focused support for vulnerable groups are needed to improve chronic disease management.

## 1 Introduction

According to the World Health Organization (1), chronic non-communicable diseases accounted for 41.1 million of the total number of deaths in the world, accounting for 7 in 10 deaths. The world population is aging, and the frequency and mortality of common chronic diseases such as hypertension, diabetes, asthma, and chronic obstructive pulmonary disease are rising sharply, threatening the quality of human life. The emergence of chronic diseases in China is also a major problem. According to the Seventh National Bureau of Statistics, the number of older adults in China over 60 years old in 2010 was 264 million, accounting for 18.7%of the total population (2), and the number is expected to increase to 255 million by 2030. This is a huge pressure on the health system. Among them, over 180 million older adults individuals live with chronic non-communicable diseases, which account for 88.5% of deaths and 69.7% of disease burden (3, 4). The medical-dominated chronic disease management model has difficulty meeting the long-term health needs of individuals. Therefore, self-management is a strategic measure to address the growing chronic disease challenge.

Chronic Disease Self-Management Behavior (CDSMB) refers to the process in which people, with the help of medical professionals, carry out preventive or therapeutic health activities. It specifically covers aspects such as disease awareness, symptom tracking, compliance with drug regimens, adoption of a healthy lifestyle, and rational use of medical services (5–7). These practices can help people cope with the physical and psychological stress caused by chronic diseases in their daily lives, and play a crucial role in preventing long - term health problems (8), slowing down or even preventing disease progression, and improving health - related quality of life (9). However, the poor physical condition caused by chronic diseases, the cognitive and emotional disorders of chronic disease patients themselves, as well as external environmental factors, make the implementation of self-management behaviors more difficult (10). Therefore, it is of great importance to clarify the factors influencing the success of chronic disease self-management.

The status of regional medical resources is an important factor affecting the self-management of chronic disease patients, especially in areas with scarce medical resources (11). A study in Australia has confirmed that poverty and medical accessibility directly affect the self-management confidence of chronic disease patients. People in areas with poor medical resources and conditions show worse self-management behaviors (12). This imbalance in the distribution of medical resources is particularly evident at the prefecture-level city level. As areas with a high-density population, the richness and rationality of medical resources in cities are directly related to the management effectiveness of many chronic disease patients. In some big cities, there are many large-scale general hospitals and specialized hospitals, equipped with advanced medical equipment, professional medical teams, and rich medical resources. Patients can easily obtain comprehensive diagnosis and treatment services, which provides a solid foundation for the self-management of chronic diseases (13). Patients can get accurate diagnoses, professional treatment advice, and regular follow-up guidance in a timely manner, which helps them better master disease knowledge and enhance their self-management ability and confidence (14).

However, in sharp contrast, the medical resources in many small and medium - sized cities and remote areas are extremely scarce. More than 40% of the world’s population cannot reach a medical institution within 1 h. People living in rural or remote urban areas often have to face longer travel times, higher medical costs, and worse medical environments (15). These regions may lack professional chronic disease diagnosis and treatment departments, have obsolete medical equipment, and a shortage of professional doctors. Even the basic examinations and treatments for common chronic diseases are difficult to guarantee. In such a situation, patients not only have difficulty obtaining timely and effective treatment, but also have extremely limited knowledge of chronic diseases, making self-management almost impossible (16). This huge gap in the distribution of medical resources leads to significant differences in the starting points of chronic disease management for patients in different cities. This gap is even more severe, pushing patients in cities with scarce medical resources into a more unfavorable health cycle. Recent studies have shown that strengthening the construction of medical infrastructure can reduce the complications caused by chronic diseases that have not been effectively managed (17). The number of doctors per thousand people is a core indicator for measuring the accessibility of regional medical care, which is directly related to the convenience of individuals in obtaining professional medical guidance. At the prefecture-level city level, the level of this indicator not only reflects the richness of medical resources, but is also a factor affecting self-management behaviors. In cities with rich medical resources, patients can more easily communicate with healthcare providers and obtain personalized self-management plans, which greatly reduces the difficulty and uncertainty of self-management (18).

The successful management of chronic diseases also depends on the daily perseverance of patients. However, the reality is that about 50% of patients do not consistently follow the prescribed treatment plans, including drug taking, diet control, and physical exercise (19, 20), which directly leads to the progression of the disease and the occurrence of long-term complications. Many factors at the individual level play a key role. Previous studies have indicated that health literacy is closely related to chronic disease self-management behaviors. A systematic review shows that high health literacy is closely related to better diabetes management outcomes, including blood sugar control, disease knowledge, and self-management ability (21). Bandura’s self-efficacy theory points out that self-efficacy (SE) is one of the core variables affecting self-management practices (22, 23). Self-efficacy is closely related to better self-management, such as blood pressure reduction, blood sugar level improvement, and better medication compliance (24, 25). This internal belief can help patients avoid being overwhelmed by the management of chronic diseases and encourage them to adhere to healthy living habits in the long-term. In addition, family health (FH) plays an indispensable role in providing basic medical care and has a close relationship with chronic disease self-management behaviors (26).

At present, there have been some studies on chronic disease self-management behaviors. However, many of these studies only focus on the individual level, exploring the relationship between the characteristics of chronic disease patients themselves and their self-management behaviors, while ignoring the multi-level structure of the data, that is, the role of higher-level prefecture-level city level factors (27–30). In addition, most of these studies focus on the characteristics of a single chronic disease or a specific population, and there are few studies on the overall chronic disease population. Based on the above-mentioned research limitations, this paper uses multi-level regression analysis to explore the influencing factors of chronic disease self-management behaviors at the individual level control variables and prefecture-level city level factor, and further analyzes the interaction between individual control variables and prefecture-level city level factor, to provide a basis for policymakers to formulate targeted prevention and control strategies.

Based on the aforementioned research gaps and theoretical foundations, this study aims to explore the multi-level influencing factors of CDSMB and proposes the following research hypotheses: H1: Health literacy positively predicts CDSMB. H2: Self-efficacy positively predicts CDSMB. H3: Family health status positively predicts CDSMB. H4: The number of physicians per 1,000 population is a cross-level positive predictor of CDSMB among patients with chronic disease.

## 2 Methods

### 2.1 Study setting and participants

The data of this study were derived from the “Psychology and Behavior Investigation of Chinese Residents (PBICR)” conducted from July 10 to September 15, 2021 (31, 32). This survey adopted a multi-stage sampling method and was carried out in 31 provinces/autonomous regions/municipalities directly under the Central Government in mainland China (including 23 provinces, 5 autonomous regions, and 4 municipalities directly under the Central Government, excluding Hong Kong, Macao, and Taiwan regions), ultimately covering 120 sample cities. The sampling process was divided into three stages: first, city sampling, where all provincial capital cities were intentionally included, followed by the random selection of 2–6 non-provincial capital cities within each province or autonomous region (the number of cities selected was linked to the population proportion of the province), resulting in the identification of 120 sample cities in 2021; second, community sampling, which took these 120 sample cities as the scope, followed the Probability Proportional to Size (PPS) principle, and used a random number table to sample 6–36 communities in each city (the number of communities was determined based on the population size of the city), ultimately selecting approximately 780–800 sample communities; third, resident sampling, which took the sample communities as the scope, referred to the data of the 7th National Population Census in 2021, implemented quota sampling based on three attributes: “gender, age, and urban–rural distribution,” and assigned sampling quotas to each community to ensure that the demographic characteristics of the sample were consistent with the national population distribution, with a total of 11,031 participants included in the survey (including 5,033 males and 5,998 females) (33).

Inclusion criteria were: (1) diagnosed with chronic diseases. (2) residents of mainland China and holding permanent residency status, identifying as ethnically Chinese, with annual travel limited to ≤1 month. (3) age 18 years and above. (4) Willingness to participate as a volunteer in the study and provide informed consent. (5) The capacity to independently finish online or paper questionnaire surveys, along with a clear comprehension of the questionnaire content. After data cleaning and excluding participants who did not meet the inclusion criteria, a total of 1,916 participants were finally included in the study.

### 2.2 Assessment instruments and evaluation

#### 2.2.1 Physicians per 1,000 population

In this study, the number of physicians per 1,000 population at the prefecture-level city was calculated as: using the year-end permanent population from the “China Statistical Yearbook 2021” as the base, data on licensed physicians and licensed assistant physicians were primarily obtained from provincial 2021 statistical yearbooks (licensed doctors and assistant doctors refer to practicing medical personnel holding corresponding licenses, excluding those in management, categorized into clinical, traditional Chinese medicine, stomatology, and public health); missing data were supplemented from local prefecture-level cities’ “Statistical Communique on National Economic and Social Development,” with the final indicator computed via the formula: (number of licensed doctors + number of licensed assistant doctors) / year-end permanent population × 1,000.

#### 2.2.2 Chronic Disease Self-Management Study Measures (CDSMS)

The Chronic Disease Self-Management Study Measures (CDSMS) (34) was employed to evaluate chronic patients’ self-management behavior. Composed of 15 items across three dimensions, which are Exercise dimension, Communication dimension and cognitive symptom management dimension. The scale assesses exercise via weekly duration (none = 0 to over 3 h = 4) and the other two dimensions by response frequency (none = 0 to constantly = 5). Higher scores signify better self-management, with the Chinese version demonstrating Cronbach’s *α* of 0.78–0.82.

#### 2.2.3 Short-Form Health Literacy Survey Questionnaire (HLS-SF12)

The Short-Form Health Literacy Survey Questionnaire (HLS-SF12) (35) includes 12 items across three dimensions: Health Care, Disease Prevention, and Health Promotion. Rated on a 4-level scale (1 = very difficult to 4 = very easy), health literacy index is calculated as (average − 1) * (50/3), ranging 0–50 (higher index = higher literacy). The scale has Cronbach’s *α* = 0.918.

#### 2.2.4 Self-efficacy (NGSES)

Self-efficacy was measured using the 8-item New General Self-Efficacy Scale-Short Form (NGSES) (36), a 5-point Likert scale (1 = strongly disagree to 5 = strongly agree). This study showing Cronbach’s *α* = 0.928.

#### 2.2.5 Family health (FHS-SF)

Family health was evaluated via the Short Form of the Family Health Scale (FHS-SF) (37), a 10-item 5-point Likert scale (1 = strongly disagree to 5 = strongly agree). Items 6, 9, and 10 are reverse-scored, yielding total scores (10–50) where higher values indicate better family health. The scale exhibited Cronbach’s *α* = 0.843 in this research.

### 2.3 Statistical analyses

Data analyses were performed using SPSS 21.0 for Windows and R version 4.4.3. To enhance the representativeness of the sample relative to the target population, this study adopted a post-stratification weighting strategy (38). With gender, age group, education level, and urban–rural distribution (four core demographic characteristics sourced from the National Population Census) serving as stratification dimensions, the population distribution information of these characteristics was utilized in the study to act as predictors for “failure to participate in the survey”. The weight factors for each stratified unit were calculated via the Inverse Probability Weighting (IPW) method. This approach ensured that the demographic structure of the adjusted sample was consistent with the overall distribution of the national resident population, thereby reducing the impact of sampling bias on the analysis results. In particular, the “lme4” package in R was employed to construct multilevel linear models. The associations between CDSMB scores and demographic variables were examined with independent samples t-tests and one-way analysis of variance (ANOVA) with post-hoc tests. Relationships between physicians per 1,000 population, contextual factors (health literacy, self-efficacy, family health), and CDSMB were analyzed using Pearson correlation coefficients. After controlling for significant demographic variables identified in previous analyses, three hierarchical linear models (HLMs) were constructed to account for the nested data structure. A Null Model was first established to estimate baseline variance components without any predictors. Model 1 then incorporated individual-level control variables (health literacy, self-efficacy, family health status) and covariates, followed by Model 2 which further added prefecture-level city level variable (physicians per 1,000 population) while controlling for the significant demographic influences identified earlier.

## 3 Results

Altogether, 1916 residents were invited to participate in this study (Table 1). Among them, 46.40% of the sample were females, while males accounted for 53.60%. The largest age group was ≥60 years old, comprising 36.27% of the sample, and Han nationality represented 93.95% of the population. A substantial proportion (24.79%) had a bachelor degree or above, and 77.97% were married. The majority of respondents (70.41%) lived in urban areas, with 57.99% having a monthly per capita income of 3,001–6,000 RMB. Additionally, regarding living alone, the overwhelming majority (90.24%) did not live alone. Table 2 shows the associations between the mean CDSMB score and demographic characteristics.

**Table 1:** Socio-demographic characteristics of the participants (*n* = 1,916).

Variable	*n*	%
Gender		
Male	1,027	53.60%
Female	889	46.40%
Age		
≤30	122	6.37%
31–45	430	22.44%
46–59	669	34.92%
≥60	695	36.27%
Ethnicity		
Han nationality	1800	93.95%
Other nationality	116	6.05%
Education		
Junior high school and below	818	42.69%
High school and technical secondary school	383	19.99%
Junior college	240	12.53%
Bachelor degree or above	475	24.79%
Per capita income(RMB)		
≤3,000	257	13.41%
3,001–6,000	1,111	57.99%
>6,000	548	28.60%
Marital status		
Married	1,494	77.97%
Divorced, widowed, or unmarried	422	22.03%
Urban–rural distribution		
Urban	1,349	70.41%
Rural	567	29.59%
Living alone		
Yes	187	9.76%
No	1729	90.24%
Number of chronic diseases		
1	1,252	65.34%
2	462	24.11%
3	146	7.62%
≥4	56	2.92%
	Mean	SD
Physicians per 1,000 population	3.24	0.81
Health literacy	31.48	8.16
Self-efficacy	28.22	5.28
Family health status	38.09	6.49
CDSMB	24.02	10.81
Exercise	6.04	4.98
Communication	6.53	3.47
Cognitive symptom management	11.45	5.72

CDSMB, Chronic disease self-management behavior.

**Table 2:** Univariate analyses of factors associated with severity of chronic disease self-management behavior (*n* = 1,916).

Variable	Exercise dimension		Communication dimension		Cognitive symptom management dimension		CDSMB score	
	M ± SD	t/F	M ± SD	t/F	M ± SD	t/F	M ± SD	t/F
Gender		4.916***		−0.599		2.517*		3.419***
Male	6.56 ± 5.23		6.48 ± 3.50		11.76 ± 5.97		24.80 ± 11.25	
Female	5.44 ± 4.61		6.58 ± 3.43		11.10 ± 5.39		23.12 ± 10.22	
Age		52.119***		2.492		22.289***		35.459***
≤30	8.80 ± 5.63		6.93 ± 4.01		12.67 ± 5.76		28.4 ± 12.25	
31–45	7.61 ± 5.73		6.74 ± 3.61		12.71 ± 6.07		27.06 ± 11.87	
46–59	6.11 ± 4.62		6.59 ± 3.29		11.78 ± 5.49		24.48 ± 9.93	
≥60	4.52 ± 4.11		6.26 ± 3.43		10.15 ± 5.43		20.94 ± 9.78	
Ethnicity		−0.811		0.584		0.062		−0.154
Han nationality	6.02 ± 4.98		6.54 ± 3.48		11.46 ± 5.74		24.01 ± 10.79	
Other nationality	6.41 ± 5.04		6.34 ± 3.22		11.42 ± 5.42		24.17 ± 11.28	
Education		62.259***		10.998***		18.390***		50.933***
Junior high school and below	4.44 ± 4.26		6.07 ± 3.36		10.43 ± 5.62		20.94 ± 10.03	
High school and technical secondary school	6.32 ± 4.63		6.51 ± 3.51		11.77 ± 5.84		24.60 ± 10.36	
Junior college	6.70 ± 4.95		6.80 ± 3.30		11.87 ± 5.39		25.36 ± 10.06	
Bachelor degree or above	8.24 ± 5.45		7.19 ± 3.59		12.76 ± 5.63		28.19 ± 11.24	
Per capita income(RMB)		33.337***		6.061**		10.611***		26.514***
≤3,000	4.38 ± 4.96		5.89 ± 3.18		10.31 ± 5.53		20.59 ± 10.99	
3,001–6,000	5.82 ± 4.79		6.54 ± 3.43		11.33 ± 5.75		23.69 ± 10.50	
>6,000	7.28 ± 5.08		6.80 ± 3.63		12.24 ± 5.63		26.32 ± 10.86	
Marital status		−1.710		1.757		−0.621		−0.510
Married	5.94 ± 4.74		6.60 ± 3.39		11.41 ± 5.73		23.95 ± 10.44	
Divorced, widowed, or unmarried	6.41 ± 5.73		6.27 ± 3.71		11.61 ± 5.67		24.28 ± 12.05	
Urban–rural distribution		6.458***		2.382*		3.376***		5.527***
Urban	6.51 ± 5.01		6.65 ± 3.48		11.74 ± 5.75		24.90 ± 10.80	
Rural	4.92 ± 4.72		6.24 ± 3.43		10.78 ± 5.58		21.93 ± 10.57	
Living alone		0.389		−0.834		1.753		0.838
Yes	6.18 ± 5.43		6.33 ± 3.67		12.15 ± 5.33		24.65 ± 11.17	
No	6.03 ± 4.93		6.55 ± 3.45		11.38 ± 5.75		23.95 ± 10.77	

Values in boldface indicate that the results of the t-test or F-test are statistically significant. *p < 0.05, **p < 0.01, ***p < 0.001.

As shown in Table 2, significant differences in CDSMB scores among chronic disease patients were observed across gender, age, educational level, monthly per capita income, and residential location. Specifically, male patients achieved significantly higher CDSMB scores than females, particularly in the exercise dimension and the cognitive symptom management dimension. Regarding age, patients under 30 years old had the highest CDSMB scores, while those over 60 years old had the lowest. In terms of educational level, CDSMB scores increased with the improvement of education. Patients with primary school education or below had significantly lower scores than those with high school and technical secondary education as well as junior college education, with patients holding a bachelor’s degree or above achieving the highest scores. Additionally, patients with a monthly per capita income of less than 3,000 yuan had significantly lower CDSMB scores than those with an income between 3,000 and 6,000 yuan, and also significantly lower than those with an income exceeding 6,000 yuan. Patients residing in urban areas had significantly higher CDSMB scores than those living in rural areas.

### 3.1 Descriptive statistics and correlational analyses

Table 3 illustrates the association between Physicians per 1,000 population, health literacy (HL), self-efficacy (SE), family health status (FH), and chronic disease self-management behavior (CDSMB). A total of 1,916 participants were included in the analysis.

**Table 3:** Descriptive statistics and correlation matrix (*n* = 1,916).

Variable	1	2	3	4	5
1 Physicians per 1,000 population	1				
2 Health literacy	0.102***	1			
3 Self efficacy	0.063***	0.445***	1		
4 Family health	−0.027	0.328***	0.460***	1	
5 Chronic disease self-management behavior	0.149***	0.371***	0.244***	−0.001	1

***p < 0.001.

Correlation analysis showed significant associations. The number of physicians per 1,000 population was significantly and positively associated with CDSMB (*r* = 0.149, *p* < 0.001), as were health literacy (*r* = 0.371, *p* < 0.001) and self-efficacy (*r* = 0.244, *p* < 0.001). By contrast, family health status was not significantly associated with CDSMB (*r* = −0.001, *p* > 0.05). The correlation between family health status and physicians per 1,000 population was not significant (*r* = −0.027, *p* > 0.05). Additionally, physicians per 1,000 population was significantly and positively correlated with health literacy (*r* = 0.102, *p* < 0.001) and self-efficacy (*r* = 0.063, *p* < 0.001); health literacy was significantly and positively correlated with self-efficacy (*r* = 0.445, *p* < 0.001) and family health status (*r* = 0.328, *p* < 0.001); self-efficacy was significantly and positively correlated with family health status (*r* = 0.460, *p* < 0.001).

### 3.2 Hypothesis testing: multilevel modeling

This study employed Hierarchical Linear Modeling (HLM) to construct three progressive hierarchical models for the systematic analysis of factors influencing Chronic Disease Self-Management Behavior (CDSMB), with detailed results presented in Table 4. The null model (Model 0), which included no independent variables, yielded an intercept of 25.267 (*p* < 0.001), representing the overall marginal mean of CDSMB scores across all participants with high statistical reliability. The residual variance (σ^2^ = 99.455) reflected the random variation at the individual level (Level 1), i.e., the dispersion of CDSMB scores among individuals within the same prefecture-level city. The between-group variance (*τ* = 6.292) characterized the systematic variation at the prefecture-level city level (Level 2), indicating substantial differences in the average CDSMB levels across different prefecture-level cities. In the goodness-of-fit test, the chi-square value was 36.935 (*p* < 0.001), rejecting the null hypothesis that “the between-group variance is zero” and confirming that the variation in CDSMB scores has a significant hierarchical structure. Thus, the application of HLM for hierarchical analysis is statistically reasonable and necessary, as it can effectively disentangle the independent effects of the individual and prefecture-level city levels. The model deviance was 14325.66, serving as a benchmark for subsequent model comparisons.

**Table 4:** Results of hypothesis testing.

Variable	CDSMB score		
	Null model	Model 1	Model 2
Prefecture-level main effect			
Physicians per 1,000 population			0.077*
Individual-level main effect			
Health Literacy		3.065***	3.075***
Self-Efficacy		1.904***	1.891***
Family Health Status		−1.811***	−1.784***
Individual-level control variables			
Gender	−1.361***	−0.934*	−0.946*
Age	−1.734**	−1.187***	−1.214***
Education	1.321***	0.994***	0.959***
Per capita income	1.286**	0.869*	0.858*
Urban–rural distribution	−0.172	0.915	0.976
R(Sigma_squared)	99.455	87.843	88.003
U(Tau)	6.292	4.066	3.376
Deviance	14325.66	14072.22	14067.69
CHI-square	36.935***	251.441***	259.97***

*p < 0.05, **p < 0.01, ***p < 0.001.

Model 1 incorporated individual-level core independent variables and control variables. Among the core independent variables, health literacy (*β* = 3.065, *p* < 0.001) and self-efficacy (*β* = 1.904, *p* < 0.001) exhibited significant positive predictive effects on CDSMB, indicating that each one-unit increase in these variables was associated with a significant increase in CDSMB scores by 3.065 and 1.904 units, respectively. Family health status (*β* = −1.811, *p* < 0.001) showed a significant negative predictive effect, suggesting that poorer family health status was associated with lower individual CDSMB levels. All control variables (gender, age, education level, per capita income, and urban–rural distribution) passed the statistical significance test (*p* < 0.05) and were retained in subsequent models to eliminate confounding effects. The individual-level residual variance of this model decreased to 87.843 (a 11.68% reduction compared to the null model), indicating that individual-level variables collectively explained 11.68% of the individual-level variance. The between-group variance at the prefecture-level city level was 4.066 (a 35.37% reduction compared to the null model), suggesting that individual characteristics also partially explained the systematic differences across prefecture-level cities. The chi-square value was 251.441 (*p* < 0.001), which remained significant, indicating that the remaining between-city variance was not fully explained and that prefecture-level city level variable needed to be further incorporated. The model deviance decreased significantly to 14072.22 (Deviance = 253.44, *p* < 0.001) compared to the null model, confirming that the inclusion of individual-level variables significantly improved model fit. From the above results, it can be concluded that Hypotheses H1 and H2 have been verified, while Hypothesis H3 has not been verified.

Model 2 further integrated the core prefecture-level city level independent variable (number of physicians per 1,000 population) based on Model 1. The results showed that the number of physicians per 1,000 population had a significant positive predictive effect on CDSMB (*β* = 0.077, *p* < 0.05), meaning that each one-unit increase in this indicator was associated with a significant increase in the average CDSMB score at the prefecture-level city level by 0.077 units. The direction and significance of the coefficients for all individual-level independent variables and control variables remained stable, indicating that prefecture-level city level variable did not moderate the mechanism of action of individual-level city level variables, and the effects of the two levels on CDSMB were independent. Model fit indicators revealed that the individual-level residual variance was 88.003 (essentially consistent with Model 1, which was consistent with theoretical expectations). The between-group variance at the prefecture-level city level further decreased to 3.376 (a 17.0% reduction compared to Model 1), indicating that the number of physicians per 1,000 population explained 17.0% of the remaining between-city variance. The chi-square value was 259.97 (*p* < 0.001), which remained significant, suggesting that additional potential prefecture-level city level variables (such as medical resource accessibility and health education coverage) may exist and require further exploration in future studies. The model deviance was 14067.69, a slight but significant decrease compared to Model 1 (Deviance = 4.53, *p* < 0.05), confirming that the inclusion of prefecture-level city level variables further optimized model fit and provided additional statistical explanatory power for CDSMB. From the above results, it can be concluded that Hypothesis H4 has been verified.

## 4 Discussion

This study aims to analyze the influence of the individual level control variables and prefecture factor on the self- management behavior of chronic disease patients, and explore the determinants of self- management behavior. Using the hierarchical linear model (HLM), this study analyzed the influences of individual and prefecture-level factors on the self- management behavior of chronic disease patients.

The results show that there is a significant difference in the level of self- management of the patients with chronic diseases, and that females, older adults, people with lower levels of education, people with lower incomes, and people living in rural areas have a relatively weaker self- management ability. The self- management score of chronic disease patients in this study was 24.02 ± 10.81, which is a relatively low level of self- management, and this result is consistent with the previous studies (30, 39–43).

At the prefecture-level city level, the positive predictive effect of the number of physicians per 1,000 population on CDSMB (*β* = 0.077, *p* < 0.05) carries profound policy implications. This variable reduces the between-group variance explanation rate of the multilevel model from 4.066 to 3.376, indicating that differences in access to medical resources account for approximately 17% of the variations in individuals’ health management behaviors. A plausible explanation is that sufficient primary care physician resources ensure patients receive continuous, personalized guidance. Primary care doctors can develop targeted exercise, diet, and medication plans based on patients’ conditions, helping them establish a scientific self-management framework and reducing cognitive misunderstandings and disease management pressures (44). Furthermore, areas with higher physician density significantly lower barriers to medical access (15). Particularly for older adults or mobility-impaired chronic disease patients, more convenient medical services can improve their compliance with regular re-examinations and management plan adjustments, thereby forming a positive cycle (45). An increase in primary care physician resources can enhance patients’ trust in the medical system, which translates into motivation to persist in self-management behaviors.

At the individual level control variables, health literacy (*β* = 3.075, *p* < 0.001) and self-efficacy (*β* = 1.891, *p* < 0.001) stand out as core drivers, with their positive effects aligning with findings from both domestic and international research (14, 23, 46, 47). Notably, family health status exerts an unexpected negative impact (*β* = −1.784, *p* < 0.001). This phenomenon may be linked to the tendency among individuals in China’s collectivist cultural context to overly prioritize family responsibilities, thereby neglecting their own health management (48, 49), which is consistent with conclusions drawn from some studies focusing on young patients (50). Additionally, mental health status may be a potential factor contributing to this result. Patients with chronic diseases are likely to experience a range of psychological and social issues (51–54), and these psychosocial problems may reduce the patients’ perception of support from their family members (55, 56), thereby further affecting their self-management behaviors (57).

From a policy practice perspective, this result directly demonstrates the value of China’s tiered healthcare reform. Previous studies have shown that abundant medical resources and China’s hierarchical medical system reform can significantly improve doctor-patient relationships (58), which in turn play a vital role in the self-management behaviors of patients with chronic conditions. Taking a city with a population of 100,000 as an example, increasing the number of primary care physicians by 1 per 1,000 population could theoretically improve the self-management ability of approximately 770 patients. Doctors track changes in patients’ behaviors through regular follow-ups, promptly correct deviations, and strengthen health beliefs. This finding provides empirical evidence for optimizing the allocation of medical resources, suggesting that efforts should be made to further promote the sinking of high-quality medical resources, especially to rural and primary-level areas, and address the regional imbalance in chronic disease self-management by improving access to physician resources.

This finding provides empirical evidence for optimizing the allocation of medical resources, suggesting that further efforts should be made to promote the sinking of high-quality medical resources, with a particular focus on tilting resources toward rural and primary-level areas. By improving the accessibility of physician resources, regional imbalances in chronic disease self-management can be addressed. In the future, differentiated primary care workforce strategies can be developed for different urban hierarchies. Additionally, dynamic adaptation standards for the number of physicians per 1,000 people can be formulated based on the aging rate and chronic disease prevalence of different cities, with a higher inclination coefficient for physician allocation assigned to rural and remote areas. This will ensure that policies are more targeted and operable in practice.

## 5 Limitations

Although this study identified the impacts of prefecture-level physician density and individual factors on chronic disease self-management behaviors through a multilevel model, several limitations remain. First, the cross-sectional design prevents establishing causal and temporal relationships between variables, and the findings may be confounded by time-varying factors. Second, the prefecture-level indicator “number of physicians per 1,000 population” fails to distinguish between physician types and the actual accessibility of services. It also excludes key variables that may exert mediating or moderating effects, and further research should be conducted in the future to address these gaps. Third, in terms of effect magnitude, while the number of physicians per 1,000 population has a statistically significant positive impact on chronic disease self-management behaviors, the effect size is small. This suggests that future studies should incorporate as many other prefecture-level variables as possible to enhance the explanatory power of the results. Fourth, the study found that the family health dimension significantly negatively predicts chronic disease self-management behaviors. This result, inconsistent with theoretical expectations, requires in-depth interpretation with reference to more literature to explore the underlying complex mechanisms. Furthermore, although the data on the number of physicians per 1,000 population and the data from this database both date back to 2021, they are not entirely consistent. Due to the slow changes in health human resources, these data can be regarded as being from the same time point, which may lead to deviations in the results.

## 6 Conclusion

Analysis shows higher physician density explains 17% of prefecture-level city level variance. Vulnerable groups (older adults, rural, low-SES) show poor self-management. At individual control variables, health literacy and self-efficacy positively predict CDSMB. Family health negatively links, likely due to cultural family duty priority. It is recommended that relevant authorities scale up investment in primary healthcare resources and expand the contingent of grassroots medical practitioners. Additionally, a suite of integrated interventions should be rolled out to upgrade primary care services and deliver tailored support. These efforts are intended to strengthen patients’ ability to self-manage chronic conditions, thus easing the pressure on the healthcare system.

## Data Availability

The original contributions presented in the study are included in the article/supplementary material, further inquiries can be directed to the corresponding author.
